# Physical Activity and Cardiac Self-Efficacy Levels During Early Recovery After Acute Myocardial Infarction: A Jordanian Study

**DOI:** 10.1097/JNR.0000000000000408

**Published:** 2020-11-02

**Authors:** Abedalmajeed SHAJRAWI, Malcolm GRANAT, Ian JONES, Felicity ASTIN

**Affiliations:** 1 PhD, RN, Assistant Professor, Faculty of Nursing, Applied Science Private University, Amman, Jordan; 2 PhD, Professor, Health and Rehabilitation Sciences, School of Health Sciences, University of Salford, Manchester, UK; 3 PhD, RN, Professor, School of Nursing and Allied Health, Liverpool John Moores University, UK; 4 PhD, RN, Professor, Centre for Applied Research in Health, University of Huddersfield; and Research and Development, Huddersfield Royal Infirmary, Acre Street, Huddersfield, UK.

**Keywords:** self-efficacy, acute myocardial infarction, physical activity, activPAL, accelerometer

## Abstract

**Background:**

Regular physical activity is important for patients with established coronary heart disease as it favorably influences their coronary risk profile. General self-efficacy is a powerful predictor of health behavior change that involves increases in physical activity levels. Few studies have simultaneously measured physical activity and self-efficacy during early recovery after a first acute myocardial infarction (AMI).

**Purpose:**

The aims of this study were to assess changes in objectively measured physical activity levels at 2 weeks (T2) and 6 weeks (T3) and self-reported cardiac self-efficacy at hospital discharge (T1) and at T2 and T3 in patients recovering from AMI.

**Methods:**

A repeated-measures design was used to recruit a purposive sample of patients from a single center in Jordan who were diagnosed with first AMI and who did not have access to cardiac rehabilitation. A body-worn activity monitor (activPAL) was used to objectively measure free-living physical activity levels for 7 consecutive days at two time points (T2 and T3). An Arabic version of the cardiac self-efficacy scale was administered at T1, T2, and T3. Paired *t* tests and analysis of variance were used to examine differences in physical activity levels and cardiac self-efficacy scores, respectively.

**Results:**

A sample of 100 participants was recruited, of which 62% were male. The mean age of the sample was 54.5 ± 9.9 years. No statistically significant difference in physical activity levels was measured at 2 weeks (T2) and 6 weeks (T3). Cardiac self-efficacy scores improved significantly between T1, T2, and T3 across subscales and global cardiac self-efficacy.

**Conclusions/Implications for Practice:**

Participants recovering from AMI in Jordan did not increase their physical activity levels during the early recovery phase, although cardiac self-efficacy scores improved. This may be because the increase in cardiac self-efficacy was not matched by the practical skills and knowledge required to translate this positive psychological construct into behavioral change. This study provides a first step toward understanding the complex relationship between cardiac self-efficacy and physical activity in this population. The authors hope that these findings support the design of culturally appropriate interventions to increase physical activity levels in this population.

## Introduction

Cardiovascular disease (CVD) is the most common cause of death worldwide, causing 31% of all deaths globally (total: 17.9 million per year; World Health Organization [WHO], 2017). This figure is much greater in low- and middle-income countries, where CVD is estimated to cause 82% of deaths (WHO, 2017). Cardiac rehabilitation is considered to be one of the most effective strategies to support secondary prevention in patients with CVD (Sumner et al., 2017). It has been recommended that patients recovering from acute myocardial infarction (AMI) should participate in cardiac rehabilitation within 10 days of leaving a hospital (Winzer et al., 2018). Other activities such as returning to work, driving, and sexual activity may be resumed after 6 weeks (Winzer et al., 2018).

Cardiac rehabilitation is a multifactorial intervention that is delivered in three phases by a multidisciplinary team. Cardiac rehabilitation programs aim to optimize psychosocial health, medical risk management, and lifestyle risk factor management (British Association for Cardiovascular Disease Prevention and Rehabilitation, 2017). When delivered as intended, cardiac rehabilitation can improve health-related quality of life and physiological parameters such as left ventricle ejection fraction, exercise tolerance, and coronary risk factor profile (Zhang et al., 2018).

Phase 2 of cardiac rehabilitation represents the early recovery phase that occurs between discharge from hospital and 6–8 weeks afterward (Bäck et al., 2017). From the patient perspective, research shows that the first 6 weeks after hospital discharge after AMI represent a particularly difficult transition period, often because of a fear of AMI recurrence linked to overexertion and physical activity (Astin et al., 2009).

People who undertake regular physical activity (PA) live longer and experience fewer cardiovascular events (Winzer et al., 2018). The European Society of Cardiology recommends that healthy adults should aim for at least 150 minutes a week of moderate-intensity PA, 75 minutes a week of vigorous-intensity PA, or an equivalent combination for 4–5 days a week (Ibanez et al., 2018).

Regular PA is also an important contributor to both primary and secondary cardiovascular prevention (Dibben et al., 2018). Patients with established CVD may slow the progression of coronary stenosis, improve endothelial function, and thereby improve their overall cardiovascular health and cardiorespiratory fitness by increasing their PA levels (Winzer et al., 2018). Improving cardiorespiratory fitness is a key factor to reducing the incidence of AMI complications and CVD mortality (Wasfy & Baggish, 2019). Several physiological benefits occur when sedentary time is replaced with PA such as stepping, standing, or light-intensity PA. These include an increase in high-density lipoprotein cholesterol and reductions in waist circumference and fasting insulin levels, which, in combination, reduce all-cause mortality (Del Pozo-Cruz et al., 2018). A cohort study conducted on over 30,000 patients with AMI reported that PA recorded at 6–10 weeks after AMI predicted mortality and readmissions at 1 year postdischarge (Ek et al., 2019). Data from a Swedish national registry (SWEDEHEART-registry) that studied a sample of 22,227 patients post-AMI for a mean follow-up time of 4.2 years found that increased levels of PA were related to reduced mortality post-AMI (Ekblom et al., 2018).

It is important to provide self-management support to patients recovering from AMI to help them make healthy lifestyle changes such as increasing PA levels (Dibao-Dina et al., 2018). Patients recovering from AMI need to be given tailored advice about PA on an individual basis to establish their baseline capacity (Bäck et al., 2017). This support is often provided as part of cardiac rehabilitation, and a recent review confirmed that attendees of a cardiac rehabilitation program had higher levels of PA than their control group peers (Dibben et al., 2018). However, this type of support is not routinely available.

A WHO report on CVD identified that the Middle East, which has a population of over 400 million, has a lower level of PA than other parts of the world (WHO, 2015). Approximately one third of the male adults and half of the female adults in the Middle East are physically inactive (WHO, 2015). The reasons for this are unclear.

In Jordan, conservative traditions, cultural norms, and beliefs regarding illness and recovery may contribute to low levels of PA among the general population (Barghouti et al., 2015). Moreover, Jordan lacks open spaces and parks, which Jordanian people prefer over sports facilities for engaging in PA (Barghouti et al., 2015). In Jordan, there are no cardiac rehabilitation or related structured education programs. Therefore, both the general Jordanian population and those recovering from AMI face potential obstacles to engaging in regular PA.

Cross-sectional surveys conducted in Europe have assessed self-reported PA levels in patients diagnosed with CHD. Data from over 15,000 participants showed that self-reported PA levels increased over time, with 20% of the sample reporting adequate levels of PA at follow-up (De Smedt et al., 2016). A more recent study showed similar findings (McKee et al., 2019). Self-reported PA levels in a sample of post-AMI patients improved significantly between baseline and 3 months after AMI, but almost half of the sample did not attain a PA level that matched guideline recommendations (McKee et al., 2019).

Self-report data on PA levels provide useful information about the context and types of PA but are limited because of variations in reporting and recall bias (Ekelund et al., 2011). Moreover, few studies have reported PA data on patients with AMI obtained objectively using accelerometers.

Cardiac rehabilitation has an impact on average step count. In two studies, an increase of 40% in number of steps was reported during rehabilitation compared with nonrehabilitation days (Jones et al., 2007; Savage & Ades, 2008). It is not clear if this improvement is able to be maintained after cardiac rehabilitation.

Primary outcomes of PA considered in prior research include step count, stepping time, standing time, upright time (the sum of standing and stepping time), and sedentary time (Granat, 2012; Ryan et al., 2006).

Step count per day has traditionally been measured using a pedometer. A guide to health intervention was developed to classify PA, with individuals who take fewer than 5,000 steps per day considered to have a sedentary life and individuals who take more than 10,000 steps per day considered to be active (Tudor-Locke et al., 2012). A previous study found that patients with acute coronary syndrome, heart failure, and coronary artery bypass grafting (CABG) or valve surgery after 1 year took approximately 6,000 steps per day (Thorup et al., 2016).

Time spent upright was assessed as 5.9 hours per day among an older healthy population in the United Kingdom (Fitzsimons et al., 2013) and 7.9 hours per day among healthy adults in the United States (Bassett et al., 2014). Standing time has been shown to have a dose–response association with all-cause mortality in adults, and increasing standing may alleviate the health risks of prolonged sitting (van der Ploeg et al., 2014).

Sedentary time is recognized as an independent coronary risk factor (Del Pozo-Cruz et al., 2018). Few studies have reported on this important PA parameter in patients recovering from AMI. An Australian study by Freene et al. (2018) showed that, although there was a significant improvement in exercise capacity and light-intensity PA, overall PA levels were low and sedentary behavior was high (Freene et al., 2018), indicating a need for greater emphasis on encouraging participants in cardiac rehabilitation programs to reduce their sedentary time. In addition, one report found that a healthy adult population in the United States spent an average of 16.1 hours per day sedentary (Bassett et al., 2014), whereas another report found that older adults in the United Kingdom spent an average of 18.06 hours per day sedentary (Fitzsimons et al., 2013).

Perceived self-efficacy (SE) is an important psychological construct known to influence engagement with PA among cardiac patients. General SE is defined by Bandura as “beliefs in one's capabilities to organize and execute the course of action required to produce given attainments” (Bandura, 1997, p. 3). Evidence indicates that SE is associated with increased PA levels in patients diagnosed with CVD and improved self-management, leading to positive health outcomes (Bergström et al., 2015). SE levels function in two ways in cardiac rehabilitation participants, namely, as a determinant of engagement in PA and as an outcome of participating in PA (Woodgate & Brawley, 2008).

Cardiac SE is a cardiac-specific measure of SE that reflects a person's belief in their ability to perform specific activities that form the foundation of secondary prevention such as healthy eating, maintaining physical functioning, and smoking cessation (Wang et al., 2016).

Limited research has reported on the PA levels of patients during the early stages of recovery from AMI. The increasing burden of CVD and low levels of PA in Middle Eastern populations provide a compelling rationale for the need to measure PA levels and cardiac SE in patients recovering from AMI. Thus, this study was designed to measure free-living PA levels and self-reported cardiac SE during early post-AMI recovery (6 weeks posthospital discharge) in patients with first AMI.

### Purpose

The aims of this study were to

1. determine the PA levels (step count, stepping time, standing time, upright time [the sum of stepping time and standing time], and sedentary time) at T2 and T3; and
2. measure self-reported cardiac SE levels at T1, T2, and T3.

## Methods

### Design

A repeated-measures design was used, and data were collected at three time points: hospital discharge (T1), 2 weeks after discharge (T2), and 6 weeks after discharge (T3). These time points were selected because health behaviors established during early recovery after AMI are likely to influence long-term behaviors.

### Setting

The study was carried out at Jordan University Hospital (JUH) in Amman, Jordan. JUH is a tertiary hospital with approximately 600 beds, including a 12-bed coronary care unit (CCU). JUH is one of four public cardiac centers in Jordan and conducts > 4,000 percutaneous coronary intervention (PCI) procedures per year. Patients from most cities in Jordan are referred based on contractual agreements with the Ministry of Health. JUH also receives private patients. This hospital holds 4.2% of the total number of hospital beds in Jordan and accounts for 4.1% of all hospital admissions (Ministry of Health in Jordan, 2019). In Jordan, patients with AMI are hospitalized for 3–5 days before discharge. There is no structured education or rehabilitation provided for patients during their hospital stay or after discharge.

### Sampling and Participants

The target population was composed of all eligible patients admitted to the CCU with a clinically confirmed AMI (ST-elevation MI and non-ST-elevation MI). Participants were included in the study if they could read and understand the study information, were aged ≥ 18 years, were admitted to the CCU with a confirmed diagnosis of first AMI, and were clinically stable with an ejection fraction of > 35%. Patients with significant comorbidities that impede PA behaviors as well as patients treated by CABG were excluded. A sample of 100 participants was targeted, based on a power calculation performed in a previous study and on an assumed attrition rate of 20% (Cowie et al., 2011).

### Ethical Approval

The University of Salford in the United Kingdom (HSCR14/120) and the institutional review board at JUH in Jordan (07/2015) granted ethical approval. The study was conducted according to the agreed protocol, and informed consent was sought before participation.

### Data Collection Procedure

Data were collected between March and December 2015. A clinical nurse identified and screened all potential patients using hospital records. Eligible patients were approached in the CCU and provided with the participant information sheets. Twenty-four to 48 hours later, hemodynamically stable patients were transferred to the medical cardiac unit. At this time, the researcher discussed the study with eligible, interested patients and obtained informed consent. Demographic and clinical data were collected from patient questionnaires and medical records. The activPAL3 monitor (a body-worn PA monitor) was attached to the participants in the outpatient clinic. The participants revisited the clinic 2 and 6 weeks after hospital discharge. The participants were asked to wear the activPAL3 monitor at all times (24 hours/day, 7 days/week) but were told it could be removed temporarily when exposed to water. A paper version of the Arabic version of the cardiac SE questionnaire was administered at T1, T2, and T3.

### Measures

#### Cardiac self-efficacy questionnaire

Cardiac SE was measured using the Cardiac Self-Efficacy Questionnaire (CSEQ; Sullivan et al., 1998), which is designed to evaluate specific aspects of SE relevant to coronary heart disease. This is important because SE has been strongly associated with changes in health behavior that may lead to beneficial health outcomes in patients diagnosed with CHD. This condition-specific measure, which reflects specific secondary prevention tasks, was chosen rather than a generic SE measure because there is evidence that SE beliefs that reflect specific tasks are stronger predictors of performance than generic SE beliefs (Rodgers et al., 2013). The CSEQ consists of 16 items divided into three sections: controlling symptoms (eight items), maintaining function (five items), and three items related to a healthy lifestyle (obesity, smoking, and dietary habits). Respondents are asked to rate how confident they feel on a 5-point Likert scale (0 = *not at all* to 4 = *completely confident*). The internal consistency of the CSEQ is .90 (Cronbach's alpha) for controlling symptoms and .87 for maintaining function (Sullivan et al., 1998).

To measure CSEQ in an Arabic population, this measure was translated and back-translated between English and Arabic. The forward translation into Arabic was performed independently by two Arabic-speaking researchers in the same field of research. An initial Arabic version of the CSEQ was produced based on a synthesis of these two translations. After that, a native-English-speaking researcher implemented the backward translation into English and resolved any inconsistencies. Some minor adaptations were made: Modern standard Arabic (Fusha) was used, gender applicability was changed, and the alternative response “Nonapplicable” was deleted. Face and content validity and reliability were examined, with the results showing the CSEQ as valid and reliable (Cronbach's alpha = .84).

#### Physical activity

The activPAL3 monitor is widely used to assess free-living PA, with previously established reliability and validity for step count, upright time, and sedentary time (Ryan et al., 2006). Free-living PA is defined as “the level of activity that the patients, within their physical limitations, at their own pace, and in their own environment, typically perform” (Moy et al., 2009). The activPAL3 has been cited as the gold standard for measuring sedentary behavior (Lyden et al., 2012) and has been validated in a wide range of populations such as older adults (Grant et al., 2008), in measuring posture and motion in adults (Godfrey et al., 2007), and in assessments of people with chronic heart failure (Cowie et al., 2011).

The activPAL3 is a lightweight (15 grams), small (53 × 35 × 7 mm), thigh-worn accelerometer-based device. It is attached to the skin at the midpoint of the anterior thigh using self-adhering, patented dual-layer hydrogels (PAL*stickies*). PA data are considered to be valid if data are available for a minimum of 3 days with at least 13 hours of measurement per day at each time point available for analysis (Edwardson et al., 2016).

### Data Analysis

The activPAL3 data were downloaded using the manufacturer's software (ActivPAL Professional Software Version 7.2.32). Data were cleaned to remove nonwear periods (Edwardson et al., 2016). Data related to stepping, standing, and sedentary duration and number of steps were extracted. Upright time was calculated by adding standing and stepping time. All measures were reported as average per day. CSEQ questionnaire data were extracted from paper-based surveys. CSEQ data and activity outcome data were transferred to the IBM SPSS Version 22 (IBM, Inc., Armonk, NY, USA). Descriptive statistics, paired *t* tests, or Wilcoxon signed rank tests (if the necessary assumptions were not met) were used to compare mean scores at two time points, and a repeated-measures analysis of variance was used to compare three or more time points. An alpha of .05 was used as the cutoff for significance.

## Results

**Figure 1.**
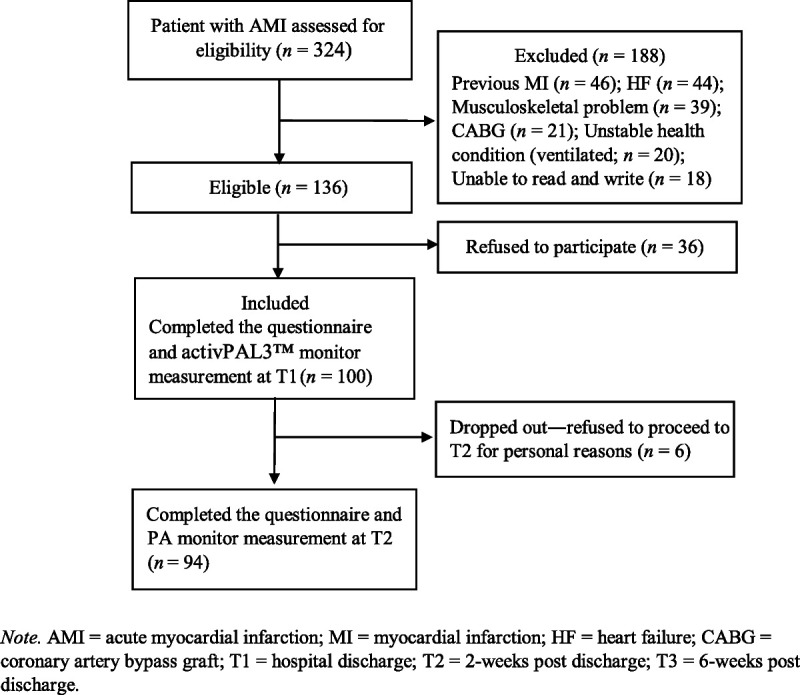
Participant Flowchart

An overview of the data collected at T1, T2, and T3 is presented in Figure 1. Of the 136 eligible patients, 36 declined to participate for personal reasons. Thus, 100 participants were initially enrolled in this study. The final sample was composed of 94 participants who were predominantly male (62%) with a mean age of 54.5 ± 9.9 years (*n* = 100) and an age range of 36–75 years (Table 1). The sample included 46 participants who had ST-elevation MI treated with primary PCI, 22 participants who were treated with thrombolytic agent (THROMB) and PCI, and 32 participants who were treated with PCI.

**Table 1.** Sociodemographic and Clinical Characteristics (*N* = 100)

Variable	*n*	%
Age (years; *M* and *SD*)	54.5	9.9
Range	36–75	
Gender		
Male	62	62
Female	38	38
Marital status		
Single/widowed	26	26
Married	62	62
Divorced	12	12
Educational level		
Secondary school or less	13	33
Higher diploma or less	21	21
Bachelor's degree	39	39
Master's degree or higher	7	7
Employment		
Employed	31	31
Unemployed	11	11
Retired	22	22
Self-employed	36	36
Clinical data		
Type of treatment		
ST-elevation myocardial infarction treated by PCI	46	46
THROMB and PCI	22	22
PCI	32	32
Diagnosis date of coronary heart disease (months; *M* and *SD*)	65.3	37.3
Range	12–216	
Body mass index (*M* and *SD*)	25.6	1.61
Range	22.0–28.5	

***Note.*** PCI = percutaneous coronary intervention; THROMB = Thrombolytic agent.

### Self-Efficacy

Table 2 shows global and subscale cardiac SE scores. There was a statistically significant increase in mean self-reported global cardiac SE scores across the three time points (22.1 ± 8.2 vs. 35.0 ± 9.1 vs. 48.0 ± 8.5). This trend was replicated across the three subscales of control symptoms (11.1 ± 4.0 vs. 16.8 ± 4.9 vs. 23.6 ± 4.6), maintain function (6.8 ± 3.0 vs. 11.2 ± 3.0 vs. 15.5 ± 2.9), and healthy lifestyle (4.3 ± 2.3 vs. 7.0 ± 2.1 vs. 8.8 ± 2.0; Table 2). There was no difference found in SE by gender, educational level, marital status, or age (*p* > .05).

**Table 2.** Cardiac Self-Efficacy at Baseline, Week 2, and Week 6: Mean and *SD* Score

Questionnaire/Subscale	Baseline (*n* = 100)		Week 2 (*n* = 100)		Week 6 (*n* = 94)		*F*	*p*
	*M*	*SD*	*M*	*SD*	*M*	*SD*		
Global Cardiac Self-Efficacy Questionnaire (16 items overall)	22.1	8.2	35.0	9.1	48.0	8.5	268.5	< .001
Subscale 1 (control symptoms)	11.1	4.0	16.8	4.9	23.6	4.6	222.6	< .001
Subscale 2 (maintain function)	6.8	3.0	11.2	3.0	15.5	2.9	224.5	< .001
Subscale 3 (healthy lifestyle)	4.3	2.3	7.0	2.1	8.8	2.0	124.6	< .001

### Physical Activity

Findings relating to PA level, including step count, stepping time, standing time, upright time, and sedentary time, are shown in Table 3. No statistically significant differences were found between T2 and T3 in terms of step count (6,819 ± 2,926 vs. 7,066 ± 2,580 steps/day) or stepping time (1.57 ± 0.64 vs. 1.62 ± 0.54 hours/day). There was a trend toward a decrease in standing time (3.98 ± 1.69 vs. 3.84 ± 1.45 hours/day) and upright time (5.55 ± 2.1 vs. 5.46 ± 1.7 hours/day) and an increase in sedentary time (18.44 ± 2.1 vs. 18.53 ± 1.74 hours/day) recorded at T2 and T3, but these differences were not statistically significant.

**Table 3.** Physical Activity Level Among Patients With AMI at Weeks 2–6

Physical Activity Measurement	Week 2 (*n* = 100)		Week 6 (*n* = 94)		*t*	*p*
	*M*	*SD*	*M*	*SD*		
Step count per day (average steps/day)	6,819	2,926	7,066	2,580	-0.55	.503
Stepping time (hours/day)	1.57	0.64	1.62	0.54	-0.54	.590
Standing time (hours/day)	3.98	1.69	3.84	1.45	0.51	.284
Upright time (hours/day)	5.55	2.10	5.46	1.70	0.30	.366
Sedentary time (sitting/lying time; hours/day)	18.44	2.10	18.53	1.74	-0.28	.380

***Note.*** AMI = acute myocardial infarction.

## Discussion

This was the first study to report on both self-reported cardiac SE and objectively measured free-living PA levels in patients with AMI during early recovery in Jordan. Mean global cardiac SE scores and associated subscale scores were found to increase significantly over time. However, this trend was not reflected in the objectively measured PA levels, which did not change significantly between T1 (2 weeks) and T2 (6 weeks).

### Cardiac Self-Efficacy

Cardiac SE is considered to be an important factor associated with behavioral change in patients with CVD (Brouwer-Goossensen et al., 2018). There are few published studies available for comparison that measure SE levels during the early recovery period (up to 6 weeks postdischarge) in patients with CHD who are not offered cardiac rehabilitation. Most prior research has focused on long-term recovery.

In this study, patients with AMI who had not attended cardiac rehabilitation showed significant improvements in global, self-reported cardiac SE, and all subscales. This is consistent with one study conducted in the United States on a sample of patients with CHD that found a statistically significant improvement in general SE recorded 2 months post-AMI (Blanchard et al., 2006). However, Blanchard and his colleagues focused on gender-based differences in SE level among patients with CHD and patients recovering from CABG and did not measure changes in SE level between hospital discharge and 2 months postdischarge (Blanchard et al., 2006).

In addition, the degree of change in SE is affected by the measure that is used. In one U.S.-based study, exercise SE levels were evaluated in 133 participants attending cardiac rehabilitation programs. Results showed that this construct was at its highest at the beginning of cardiac rehabilitation, decreased significantly 6 months later, and then had leveled off at the 18-month follow-up (Howarter et al., 2014).

Salari et al. (2016) measured cardiac SE and reported on patients recovering from angioplasty who had low cardiac SE levels at 6 months postoperation. The mean cardiac SE score was 8.43 ± 4.5, with scores ranging from 0 to 20. These findings diverge significantly from this study, which reported a mean cardiac SE score of 48.0 ± 8.5 and a score range from 0 to 64. Another study showed that post-CABG, angioplasty, and AMI patients reported lower and more-stable general SE levels over a 2-month period after cardiac rehabilitation than their control group peers (Poortaghi et al., 2013). However, this particular difference with this study may be because of the different SE measures used (Rodgers et al., 2013), different populations surveyed, and differences in measurement times. SE is a complex psychological construct that is influenced by other mechanisms that must be taken into account when comparing findings.

### Physical Activity

This was the first study to report on objectively measured PA in patients with AMI recorded during the early recovery period. No significant differences were found in any of the five PA parameters between T2 (Week 2) and T3 (Week 6). The low levels of PA found in this study support the findings of the WHO regarding the rising prevalence of CVD risk factors and the low general level of PA in Middle Eastern countries (WHO, 2016).

The mean step count per day in this study was similar to the findings of other studies on patients with AMI and ACS (Houle et al., 2011; Izawa et al., 2006) that showed patients with AMI taking approximately 6,000 steps per day at 3–6 months after cardiac rehabilitation. Thorup et al. (2016) reported 8,000 steps per day as representative of the PA guidelines for patients with CHD. The population in this study tended toward the less-active end of this range at both T2 and T3, and mean step count per day was below the recommended PA level.

Comparing objectively measured PA levels among healthy adults, patients with AMI, and older adult populations (> 65 years old), the step count in this study was below the step count of older adults reported in a previous study (7,066 steps/day vs. 8,493 steps/day), respectively. In addition, stepping time, standing time, and upright time in our sample were all below the results obtained from a sample of older, healthy adults (1.62 vs. 1.75 vs. 1.9 hours/day, 3.84 vs. 4.19 vs. 6.0 hours/day, and 5.46 vs. 5.94 vs. 7.9 hours/day, respectively). Moreover, sedentary time in the current study was higher than older adults and healthy adult populations (18.53 vs. 18.06 vs. 16.1 hours/day), respectively.

Standing time has a dose–response association with all-cause mortality in adults, and increasing standing may alleviate the health risks of prolonged sitting (van der Ploeg et al., 2014). This study found that mean upright time per day (5.46 hours) represented about 23% of the day of the participants, with sedentary time accounting for the remainder (77%, 18.53 hours). This was lower than the 32.9% reported for healthy adults in the United States (Bassett et al., 2014) and the 24.95% reported for older adults in the United Kingdom (Fitzsimons et al., 2013). Therefore, although the average age of the participants in this study was 54.5 years, their mean PA level was closer to that of the elderly population surveyed in Fitzsimons et al. (2013). Upright time did not change over the measured period.

Most time spent by the participants with AMI was sedentary, with 77% overall mean sedentary time reported at T2 and T3. Bassett et al. (2014) found that healthy adults spent 67% of their time in a sedentary state (sitting/lying time), whereas a similar study found that older adults (average: 68 years) spent 75% of their time in a sedentary state (Fitzsimons et al., 2013). This may be because of a lack of awareness of the importance of PA and a lack of structured education for patients with AMI in Jordan (Eshah, 2011). Excessive time spent in a sedentary state increases the risks of diabetes, obesity, dyslipidemia, and premature mortality (Winzer et al., 2018).

A study on the PA levels of healthy adults conducted in Jordan using a self-administered PA questionnaire provides an interesting comparison with the findings of this study (Barghouti et al., 2015), showing that PA levels were similarly low in both studies. The authors reported that a lack of awareness about the benefits of PA combined with barriers related to social and cultural norms may explain their findings (Barghouti et al., 2015). Another study conducted even more recently in the Middle East found that the healthy adult population spent most of their time either sedentary or at reduced levels of activity, finding that barriers to PA were social, cultural, and environmental as well as lack of motivation, lack of intention, and lack of awareness regarding the importance of PA (Sharara et al., 2018). These factors may help explain the lack of change in PA levels between T2 and T3 in this study. However, it has been shown that, even when interventions targeting behavioral change are introduced, obtaining meaningful and sustained changes in PA level is difficult (Greaves et al., 2011). Whatever the explanation, it is clear that there is an urgent need to increase PA levels in Middle Eastern populations.

Cardiac rehabilitation programs have been shown to increase the awareness and knowledge of patients regarding controlling CVD risk factors, improving self-management skills, and promoting PA (Dibben et al., 2018). In this study, the participants did not have access to a cardiac rehabilitation program. Thus, the findings show the need for an effective strategy to reduce sedentary time and increase PA. Therefore, patients with AMI must understand and appreciate that the need to promote their PA level is not optional but rather a necessary part of their recovery. In addition, establishing cardiac rehabilitation programs is essential for the current and future health of patients in Jordan.

This study aimed to measure the changes in SE and PA in the early recovery period. In this study, SE increased but PA did not change. However, behavioral change is a complex process that is affected by many factors, including motivation, opportunity, and capability, each of which may interact and influence the process (Michie et al., 2011). It might be that a lack of some of these factors was responsible for this observation.

This study was affected by several limitations. First, the research was conducted at a single center, which, although a major medical center with a countrywide catchment area, may limit the generalizability of the findings. Second, the sample was purposive, and > 50% of screened patients were ineligible because they had an ejection fraction < 35% and a previous AMI, were unable to read and write, or were treated with CABG. Moreover, 30% of the eligible patients with AMI refused to participate. A primary strength of this study is the objective measurement of a range of key PA parameters of a relatively large sample size with a high compliance rate, which has not been reported to date in this population.

### Conclusions

Participants recovering from AMI in Jordan did not increase their PA levels during the early recovery phase, although cardiac SE scores improved. This may be because the increase in cardiac SE was not matched by the practical skills and knowledge required to translate this positive psychological construct into behavioral change. This study was conducted in a setting where there is an absence of cardiac rehabilitation facilities, limited lifestyle intervention options, barriers to PA, and a widespread lack of knowledge regarding recommended PA levels. This study provides a first step to understanding this complex relationship and, in combination with further research, may be used to support the design of culturally appropriate interventions to increase PA levels in the target population. Further studies to understand the mechanism that increases cardiac SE among patients with AMI after hospitalization in Jordan are warranted. In addition, replication of this study with long-term measurements in different populations to confirm the results is recommended.
